# Impact of Modifications from Potassium Hydroxide on Porous Semi-IPN Hydrogel Properties and Its Application in Cultivation

**DOI:** 10.3390/polym16091195

**Published:** 2024-04-25

**Authors:** Huynh Nguyen Anh Tuan, Bui Thi Cam Phan, Ha Ngoc Giang, Giang Tien Nguyen, Thi Duy Hanh Le, Ho Phuong

**Affiliations:** 1 Faculty of Chemical and Food Technology, Ho Chi Minh City University of Technology and Education, No. 1, Vo Van Ngan Street, Linh Chieu Ward, Thu Duc, Ho Chi Minh City 71307, Vietnam; buithicamphan98@gmail.com (B.T.C.P.); giangnt@hcmute.edu.vn (G.T.N.); duyhanhle@hcmute.edu.vn (T.D.H.L.); hophuong@hcmute.edu.vn (H.P.); 2 Faculty of Chemical Technology, Ho Chi Minh City University of Industry and Trade, No. 140, Le Trong Tan Street, Tay Thanh Ward, Tan Phu District, Ho Chi Minh City 72009, Vietnam; hagn@huit.edu.vn

**Keywords:** semi-IPN hydrogel, modified hydrogel, *N*,*N*′-dimethylacrylamide, phosphate fertilizer, mustard green

## Abstract

This study synthesized and modified a semi-interpenetrating polymer network hydrogel from polyacrylamide, *N*,*N*′-dimethylacrylamide, and maleic acid in a potassium hydroxide solution. The chemical composition, interior morphology, thermal properties, mechanical characteristics, and swelling behaviors of the initial hydrogel (SH) and modified hydrogel (SB) in water, salt solutions, and buffer solutions were investigated. Hydrogels were used as phosphate fertilizer (PF) carriers and applied in farming techniques by evaluating their impact on soil properties and the growth of mustard greens. Fourier-transform infrared spectra confirmed the chemical composition of SH, SB, and PF-adsorbed hydrogels. Scanning electron microscopy images revealed that modification increased the largest pore size from 817 to 1513 µm for SH and SB hydrogels, respectively. After modification, the hydrogels had positive changes in the swelling ratio, swelling kinetics, thermal properties, mechanical and rheological properties, PF absorption, and PF release. The modification also increased the maximum amount of PF loaded into the hydrogel from 710.8 mg/g to 770.9 mg/g, while the maximum % release of PF slightly increased from 84.42% to 85.80%. In addition, to evaluate the PF release mechanism and the factors that influence this process, four kinetic models were applied to confirm the best-fit model, which included zero-order, first-order, Higuchi, and Korsmeyer–Peppas. In addition, after six cycles of absorption and release in the soil, the hydrogels retained their original shapes, causing no alkalinization or acidification. At the same time, the moisture content was higher as SB was used. Finally, modifying the hydrogel increased the mustard greens’ lifespan from 20 to 32 days. These results showed the potential applications of modified semi–IPN hydrogel materials in cultivation.

## 1. Introduction

Agriculture plays an important role in providing and ensuring food security for humanity. In farming techniques, watering and fertilizing are mandatory requirements to ensure plant growth and improve product quality and production yield. The conventional methods used to supply water and nutrients to the soil, such as spraying or spreading, cause a significant amount of fertilizer and water to be lost through volatilization and leaching, increasing agricultural costs and leading to environmental pollution [1,2]. Especially in arid climates, water scarcity is an extremely serious environmental problem due to low rainfall, which is hindering the sustainable development of agriculture. Therefore, finding a solution to properly distribute water and fertilizer plays an important role in maintaining the quality of the soil and improving the quality of the harvest. Recently, superabsorbent hydrogels (SAHs) and controlled release fertilizers (CRFs) are gradually becoming potential materials used in agricultural and horticultural areas to reduce irrigation frequency and improve soil physical properties [3,4,5].

Hydrogels are three-dimensional polymeric materials that can absorb a large amount of water and other agents while maintaining their structural integrity [6,7,8]. Absorbed agents are retained and released slowly under osmotic pressure [9]. Based on these valuable characteristics, hydrogels have been applied in many fields, such as wound healing [10], drug delivery [11], tissue engineering [12], skin care [13], hair care [14], soil conditioning [15,16], seed coating [17,18], and smart agriculture [19]. For applications of SAHs and CRF, the material must meet the requirements of high absorption capacity, controlled absorption and release of reagents, thermal stability, and high mechanical properties.

In general, hydrophilic hydrogels are often synthesized from acrylamide and its *N*-substituted monomers, such as poly(*N*-isopropylacrylamide) (PNIPAM), poly(*N*-cyclopropyl-acrylamide) (PCPA), poly(*N*,*N*′-diethylacrylamide) (PDEA), and poly(*N*-ethylacrylamide) (PEAM) [2,20,21,22]. Among them, the most popular is polyacrylamide (PAM) due to its high activity, ease of synthesis, high hydrophilicity, and being a low-cost material. However, acrylamide is classified as probably carcinogenic to humans when serious effects of acrylamide on animals have also been reported, including genotoxicity, teratogenicity, neurotoxicity, carcinogenicity, and damage to reproductive functions [23]. Therefore, limiting the use of acrylamide and finding another material to replace it in the synthesis of hydrophilic hydrogels is an urgent requirement. In this case, the other hydrogels based on *N*,*N*′-dimethylacrylamide (DMA) can be a new attractive candidate for superabsorbent applications because the cytotoxicity of PDMA-based hydrogels is non-toxic [24]. On the other hand, conventional hydrogels have several disadvantages, such as brittleness, low swelling ratios, uncontrolled absorption and release, and poor mechanical properties that hinder their reuse [25]. In comparison with the PAM-based hydrogel, the swelling ratios of the PDMA-based hydrogel were usually much lower because the chemical structure of the DMA monomer has two hydrophobic methyl -CH_3_ groups [26,27]. In our previous reports, semi-IPN hydrogels were synthesized and solved the disadvantages of conventional hydrogels [8,11,28].

In general, semi-IPN hydrogels are synthesized by simultaneous polymerization of a designed monomer mixture in the presence of a cross-linking agent and linear polymer chains that will be physically entangled within the resulting three-dimensional network. Depending on the expected physicochemical properties of the obtained hydrogels, the raw materials can be designed with different compositions. For example, using hydrophilic linear polymers leads to increased swelling ratios and the ability to control the absorption/release behaviors of semi-IPN hydrogels. Meanwhile, carboxylic acid co-monomers can introduce hydrophilic functional groups and pH-sensitive segments inside hydrogels [8,11,28]. In this work, except DMA used as a main raw material, linear PAM and maleic acid were used as a linear polymer and co-monomer. PAM is a highly hydrophilic and water-soluble polymer [29], and PAM-based hydrogel possesses excellent characteristics for SAH applications [30]. Similarly, maleic acid (MA) is an organic acid with two hydrophilic functional groups, so it can enhance hydrophilicity, improving compatibility with other hydrophilic segments by forming hydrogen bonds, and impacting the resulting hydrogels [31].

Modification is the creation of a change in the composition or structure of a material to achieve the desired characteristics. It was reported that, when modified in a KOH solution, a porous structure was created in the hydrogel. Modified hydrogels have been used for specialized applications, such as tissue engineering scaffolds, carriers of solvents, nutrients, and other agents [26,32].

As mentioned above, fertilizer is any material applied to soil or plant tissues to provide nutrients and ensure plant growth. For modern agricultural practices, fertilization focuses on three main nutrients: nitrogen (N), phosphorus (P), and potassium (K). Phosphorus is an essential nutrient and plays an important role in biochemical processes necessary for the healthy growth of plants [33]. This nutrient promotes root elongation and branching, allowing plants to access more water and nutrients from the soil [34,35]. By fertilizing with phosphorus, it can be ensured that plants are adequately supplied with phosphorus, promoting healthy growth, increasing yields, and improving crop quality. Therefore, the study of phosphorus-based CRFs can provide additional information in the field of cultivation.

In this work, a semi-IPN hydrogel (SH) was synthesized by free radical polymerization of DMA and MA monomers in the presence of the linear polymer PAM and the MBA cross-linker. Then, the SH hydrogel was modified in a KOH solution to become a modified hydrogel (SB). The chemical structures, morphology, thermal properties, rheological, and mechanical properties of polymers were investigated using FTIR, SEM, DSC, and TGA. In addition, the swelling behaviors of hydrogels were studied in water, salt solutions, and buffer solutions by the weighing method. The absorption and release of phosphate fertilizer (PF) by the hydrogels, as well as the impact of the PF-hydrogels on soil characteristics, were also evaluated. Finally, PF-hydrogels were applied in agricultural technology by evaluating their impact on the growth of mustard greens. In addition, DMA-based hydrogels possess antioxidant and antibacterial properties, which is a very important premise for finding their versatile applications [36,37]. To the best of our knowledge, this is the first time the linear polymers PAM, DMA, and MA were combined in a semi–IPN hydrogel modified with KOH and applied as a phosphorous fertilizer carrier compared to previous conventional hydrogels [26,38].

## 2. Materials and Methods

### 2.1. Materials

*N*,*N*’-dimethylacrylamide (DMA; C_5_H_9_NO; 99%) and maleic acid (MA, C_4_H_4_O_4_, 99%) were supplied by Aldrich Chemical Corp. (Saint Louis, MO, USA); polyacrylamide (PAM; C_3x_H_5x_N_x_O_x_; 99%; M_w_ = 2 × 10^6^–14 × 10^6^ g·mol^−1^) was supplied by Shanghai Macklin Biochemical Technology Co., Ltd. (Pudong, Shanghai, China); ammonium persulfate (APS, (NH_4_)_2_S_2_O_8_), as a catalyst, was purchased from Aencore Chemical Pty., Ltd. (Surrey Hills, Australia); *N*,*N*,*N*′,*N*′-Tetramethylethylenediamine (TEMED; C_6_H_16_N_2_), as a promoter, and *N*,*N*′-Methylenebisacrylamide (MBA, C_7_H_10_N_2_O_2_), as a cross-linker, was purchased from Alfa Aesar Co. (Tewksbury, MA, USA). Phosphate fertilizer (Ca(H_2_PO_4_)_2_) was supplied by Damao Chemical Reagent Factory (Dongli, Tianjin, China); sulfuric acid (H_2_SO_4_) was supplied by Aldrich Chemical Corp (Saint Louis, MO, USA), ammonium molybdate tetrahydrate ((NH_4_)_6_Mo_7_O_24_·4H_2_O), potassium antimony tartrate (K_2_Sb_2_C_8_H_4_O_12_·3H_2_O), and ascorbic acid (C_6_H_8_O_6_) were provided by Shanghai Macklin Biochemical Technology Co., Ltd. (Pudong, Shanghai, China); Lavamix Pro soil was provided by Duc Thuan Trading and Service Co., Ltd. (Ho Chi Minh, Vietnam); mustard green seeds were provided by An Phat Home Garden Co., Ltd. (Binh Duong, Vietnam). All reactants were used as received without any further purification.

### 2.2. Preparation of Semi-IPN Hydrogel

#### 2.2.1. Preparation of Semi-IPN Hydrogel

**Figure 1:**
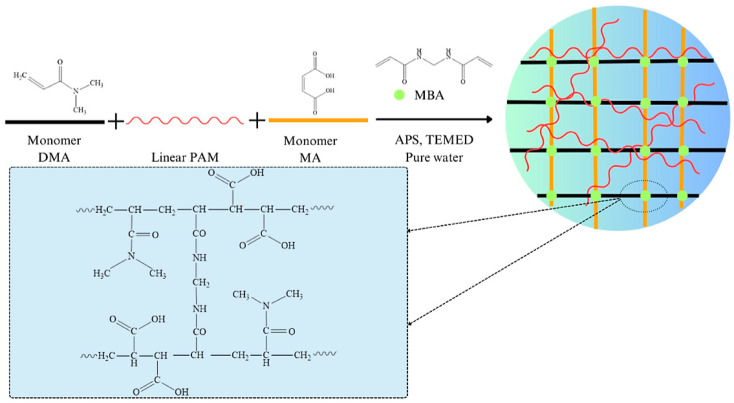
The synthesis scheme of SH hydrogel.

Semi-IPN hydrogel (SH) was synthesized by the free radical polymerization between DMA and MA in the presence of linear PAM solution; MBA was the cross-linker, TEMED was the accelerator, and APS was the initiator. The scheme of the synthesis is illustrated in Figure 1. First, 9 mL of 1% PAM solution, 9 mL DMA monomer, 1.3920 g MA, 0.12 g MBA, 7.2 mL solution of APS (0.084 mol/L), and 9 mL water were homogenized in a flask for 30 min by sonication. Then, the mixture was vacuumed for 30 min under ice-water conditions. Then, 7.2 mL solution of TEMED (1.068 mol/L) was dropped into the solution and stirred continuously for about 2 min. The mixture was rapidly poured into a mold to obtain cylindrical hydrogels with dimensions of 12 mm × 5 mm. After solidification, the hydrogels were sealed and stabilized for 24 h at 3–5 °C. Then, the hydrogel was purified with water continuously for 7 days at room temperature to remove unreacted reagents. Finally, the hydrogels were freeze-dried at −45 °C to obtain dried samples for further investigation [8,11].

#### 2.2.2. Modification of Semi-IPN Hydrogel

Immerse about 4.0 g of dried SH hydrogel into 2 L solution of KOH 2 M at 45 °C for 96 h. Then, excess KOH was removed from the modified hydrogel with water continuously for 7 days at room temperature. Finally, the swollen hydrogel was freeze-dried at −45 °C to obtain purified-modified hydrogel, named SB hydrogel [26].

### 2.3. Characterization Methods

The FTIR spectra were measured on a Jasco 4700 FTIR spectrometer (Hachioji, Tokyo, Japan) with wavenumbers in the range of 4000–400 cm^−1^ and a scan resolution of 4 cm^−1^. The ATR technique was applied directly to monomers, linear polymers, and dried hydrogels. The influence of backgrounds, such as free CO_2_ and free water, during the operation was eliminated.

A scanning electron microscope SEM (TM4000PlusII, Hitachi, Japan) was used at an acceleration potential of 10.0 kV in BSE mode to observe the morphology of the freeze-dried hydrogels. To enhance the conductivity, all hydrogels were coated with platinum. The pore size and porosity were analyzed by ImageJ software (Madison, WI, USA, https://imagej.net/ij/). In addition, the EDX spectrum was also measured to probe the potassium element of the modified-SB hydrogel using a Quantax 75 instrument (Bruker, Karlsruhe, Germany).

The glass transition temperature (T_g_) of the hydrogels was determined using a DSC 214 Polyma and Netzsch Proteus^®^ software (Netzsch, Selb, Germany, https://analyzing-testing.netzsch.com/en). Approximately 10 mg of dried hydrogel was tested at a heating rate of 5 °C/min in the temperature range of 50–220 °C under a nitrogen atmosphere. Additionally, the thermal stability of the hydrogels was evaluated using a Labsys Evo thermal gravimetric analyzer TGA (Setaram, Burladingen, Germany). The analysis was conducted at a heating rate of 10 °C/min in a temperature range of 50–900 °C under an argon atmosphere.

A Haake RheoStress rheometer (Thermo Fisher Scientific, Waltham, MA, USA) was used for oscillatory parallel-plate rheological measurements to determine the storage modulus (G′), loss modulus (G″), and viscosity (η*). These values were measured on original hydrogels as a function of time (in the range of 50–180 s), temperature of 25 °C, and frequency of 1 Hz. All tests were performed with a gap between the two discs of 3.2 mm.

The mechanical properties of the hydrogels were determined from stress-strain curves, which were measured using CT3 Texture Analyzer (AMETEK Brookfield, Middleborough, MA, USA) at 25 °C temperature. Compression tests were performed using a flat probe with a diameter of 7 mm at a speed of 0.05 mm/s on a 12 mm × 5 mm original cylindrical hydrogel until cracking. The compressive modulus values were calculated in the strain range of 10–30%.

### 2.4. Swelling Behavior

#### 2.4.1. Swelling Ratio

To investigate the swelling ratio of the hydrogels in different experimental environments, approximately 0.15 g of dried hydrogels were immersed in 100 mL of water, or 50 mM solution of salts (NaCl, CaCl_2_, AlCl_3_), or acetate buffer solutions with pH values in the range of 2–12. At specified intervals, excess water on the surface of hydrogels was removed with filter paper and weighed again to determine the weight. Then, the swelling ratio (SR) was calculated according to Equation (1) [26].

(1) $$SR=\frac{\left(M_{t}-M_{o}\right)}{M_{o}}\times 100\%$$

where M_t_ (g) is the weight of the equilibrium swelling and M_o_ (g) is the weight of the dried hydrogel.

#### 2.4.2. Swelling Kinetics

The swelling kinetics are an important characteristic of hydrogels when studying the absorption or release of agents by hydrogels. From the experimental data, the kinetic curves of the swelling as a quadratic function were calculated according to Equation (2) [26].

(2) $$\frac{dS}{dt}=k_{s}(S_{max}-S{)}^{2}$$

where dS/dt is the swelling rate, S_max_ (g_water_/g_hydrogel_) is the equilibrium swelling ratio, S (g_water_/g_hydrogel_) is the swelling ratio at t (min), and k_s_ is the swelling rate constant (g_hydrogel_/(g_water·min_).

From the above equation, the differential of S = 0 at t = 0; S = S_max_ at t = t_eq_ were obtained. Equation (3) was further derived as below with $A=\frac{1}{S_{max}^{2}};B=\frac{1}{S_{max}}$ [26].

(3) $$\frac{t}{S}=A+B\cdot t$$

Fick’s law is applied to calculate the expansion coefficient of the diffusion according to Equation (4) [26].

(4) $$F=\frac{M_{t}}{M_{e}}=k\cdot t^{n}$$

where M_e_ (g) is the equilibrium weight of the hydrogel, k is a specific rate constant, and t (min) is time.

Finally, the diffusion coefficient D (cm^2^/min) of the cylindrical hydrogel was calculated according to Equation (5) [26].

(5) $$D=\pi r^{2}{\left(\frac{k}{4}\right)}^{\frac{1}{n}}$$

where r is the radius of the cylindrical hydrogel and n is the diffusion exponent.

### 2.5. Application for Phosphate Fertilizer

#### 2.5.1. Phosphate Fertilizer Sorption Behavior

The content of phosphate fertilizer (PF) loaded on the hydrogels was determined by measuring the absorbance of the remaining PF solutions using a UV–Vis spectrophotometer (UH5300, Hitachi, Japan). Calibration curves were developed from solutions with concentrations of 2, 3, 4, 5, 10, and 20 ppm. The reagent solution was prepared by mixing the following solutions: 10 mL of 2.5 M sulfuric acid solution; 3 mL ammonium molybdate tetrahydrate solution 4 g/100 mL H_2_O; 1 mL potassium antimony tartrate solution 0.28 g/100 mL H_2_O; and 6 mL ascorbic acid solution 1.76 g/100 mL H_2_O [39]. First, approximately 0.05 g of dried hydrogel was immersed in 50 mL of PF solutions with the concentration in the range of 50–5000 ppm for 24, 48, 72, and 96 h at 25 °C. Then, 5 mL of remaining PF solution was mixed with 1 mL of reagent solution. The resulting mixture was measured for absorbance and the amounts of loaded PF per mass of dried-hydrogel (Q_e_) were determined according to Equation (6) [11].

(6) $$Q_{e}=\frac{C_{o}V_{o}-C_{t}V_{t}}{M}$$

where

▪ C_o_ (mg/L): concentration of PF solution before absorption;
▪ C_t_ (mg/L): concentration of remaining PF solution after absorption;
▪ V_o_ (mL): volume of PF solution before absorption;
▪ V_t_ (mL): volume of remaining PF solution after absorption;
▪ M (g): weight of dried hydrogel.

#### 2.5.2. Phosphate Fertilizer Release Behavior

The PF-loaded hydrogels were prepared as follows: immerse 1 g of dried hydrogels in 1 L of PF solution (4000 ppm for SH and 3000 ppm for SB hydrogel) for 72 h at 25 °C. The loaded hydrogels were dried at 45 °C to completely remove water, named PF/SH and PF/SB. The absorbance measurement was further used to determine the PF release capacity of the loaded hydrogels. The experiments were performed as follows: immerse 0.05 g of dried PF/SH or PF/SB hydrogel into 50 mL of pH4, pH8, and pH12 buffer solutions at 25 °C. At specified intervals, the pH buffer solution was measured for absorbance, and the amount of PF released was determined using the method introduced above. The PF content released into the solution was calculated according to Equation (7).

(7) $$\%Release=\frac{Q_{t}}{Q_{o}}\times 100\%$$

where Q_t_ (mg/g) is the amount of PF released at t (min) and Q_o_ (mg/g) is the initial amount of PF.

In this work, to evaluate the PF release profile, including the mechanism and influencing factors, four popular models were applied: zero-order kinetic model (Z–O) (Equation (8)), first-order kinetic model (F–O) (Equation (9)), Higuchi model (H) (Equation (10)), and Korsmeyer–Peppas model (K–P) (Equation (11)) [11]. The mathematical equations are introduced in Table 1.

### 2.6. The Impact of Hydrogels on Soil Properties

The impact of hydrogels on soil properties was evaluated through changes in moisture and pH when the swollen hydrogel was placed inside the soil. First, the dried hydrogels were immersed in water at 25 °C until they reached equilibrium. Then, the equilibrated swollen hydrogels were placed inside a soil pot containing 1 kg of soil with a dried-hydrogel/soil ratio of 0.1 g/1 kg. Hydrogels were placed in the center of the soil pot and approximately 5 cm from the top surface. At specified intervals, soil around the hydrogels was extracted to determine moisture and pH values.

The moisture of approximately 1 g of soil, which was extracted at three locations around the hydrogel, was measured using an IR moisture balance MB90 (Ohaus, Parsippany, NJ, USA) according to ASTM D2216 [40,41]. Equation (12) was used to calculate moisture W, where M_w_ (g) is the weight of water, and M_s_ (g) is the weight of dried soi. For the first cycle, moisture was measured daily until it was below 20%. For the remaining cycles, 250 mL of water was added to the soil pot before starting to investigate.

(12) $$w=\frac{M_{w}}{M_{s}}\times 100\%$$

For pH measurement, approximately 5 g of soil around the hydrogel was extracted and mixed with 25 mL of water for 30 min at 25 °C. After 15 min of stabilization, the mixture was filtered. Finally, the filtered water was measured pH using a Lab 855 Meter (SI Analytics, Mainz, Germany).

### 2.7. Plant Growth Performance

#### 2.7.1. Prepare the Plants

Immerse about 10 g of mustard green seeds into 1 L of water at 45 °C for about 4 h. After that, the immersed seeds were incubated for 24 h at room temperature. Seeds with visible sprouts were sown in soil pots with a distance of about 5–10 cm and watered in the morning and afternoon.

#### 2.7.2. Evaluate Plant Growth

First, 2.5 g of PF-dried hydrogels (PF/SH or PF/SB) were placed in a 150 mm × 160 mm pot containing 1 kg of soil. Soil and hydrogels were arranged alternately in three layers. Then, the four germinated seeds were planted in the hydrogel-soil pots, and only 500 mL of water was added at this time. The growth and length of mustard greens were observed and recorded.

## 3. Results and Discussion

### 3.1. Characterization

**Figure 2:**
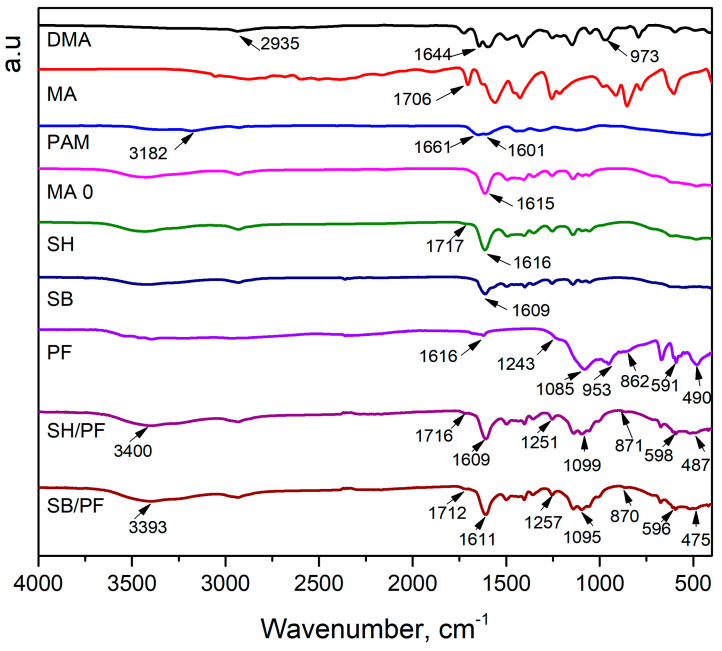
FTIR spectra of monomer, linear polymer, hydrogels, PF, SH/PF, and SB/PF.

The FTIR spectra of monomer, linear polymer, hydrogels, PF, SH/PF, and SB/PF are presented in Figure 2. The DMA spectrum exhibited vibrations of C–H peak, C=O stretching peak, CH=CH_2_ peak_,_ and C–N vibration at 2935, 1644, 973, and a range of 1050–1147 cm^−1^, respectively [42]. The characteristic functional groups of MA revealed at 1706, 1628, and 2381–3328 cm^−1^ are attributed to the C=O peak (carboxyl), C=C peak, and –OH vibrations spanning peak in order [43]. In the PAM spectrum, the three characteristic peaks of the amide group are shown at 1601 cm^−1^ (–NH_2_ bending vibration), 1661 cm^−1^ (–C=O stretching vibration), and 1415 cm^−1^ (–C≡N stretching vibration) [44]. The disappearance of the 973 cm^−1^ peak in the SH spectrum showed that polymerization occurred, and the shift of the C=O stretching peak (carbonyl) from 1644 to 1616 cm^−1^ indicated the successful incorporation of PAM into DMA and MA networks. The copolymerization of DMA and MA was demonstrated by the appearance of a 1725 cm^−1^ peak, which was attributed to the stretching vibration of C=O (carboxyl) [26]. Meanwhile, the vibration of this functional group was not observed on the SB spectrum, this is because, during the modification with KOH, the carboxyl groups were neutralized and converted into carboxylate groups.

PF consists of CaO, [H_2_PO_4_]^−^, and H_2_O in its structure. Characteristic peaks of the Ca–O bond were observed at 490, and 862 cm^−1^. The fundamental vibrational patterns of [H_2_PO_4_]^−^, including O–P–O bending, P–O stretching, in-plane P–O–H, and out-of-plane P–O–H, were attributed at 591, 1085, 1243, and 953 cm^−1^, respectively. In addition, water stains in PF were found in the range of 3400–3393 cm^−1^ [45]. The FTIR spectra of SH/PF and SB/PF contained fundamental vibrations of PF, SH, and SB which provided clear evidence of the interaction between PF and SH, as well as PF and SB, thus confirming successful loading of PF into SH and SB.

**Figure 3:**
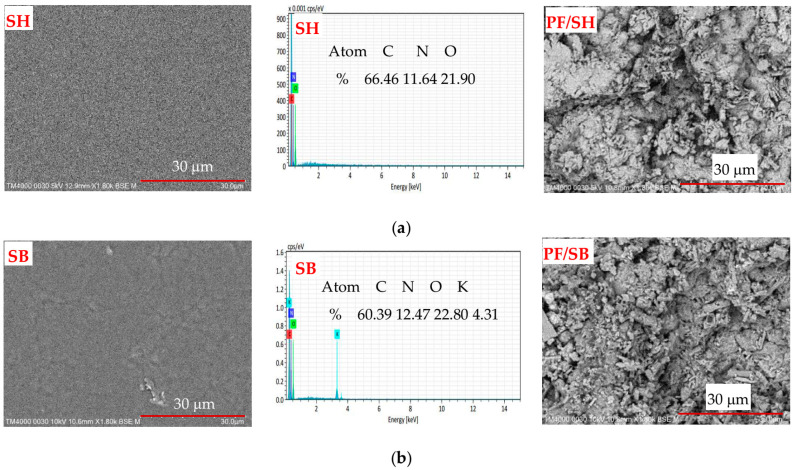
Surface morphology and EDX spectra of hydrogels, and PF-loaded hydrogels of (**a**) SH and (**b**) SB.

The surface morphology and EDX spectra of the hydrogels and PF-loaded hydrogels are presented in Figure 3a,b. The results showed that SH has a uniform and smooth surface, while SB begins to have rough spots. This difference may be due to the appearance of –COOK groups on the surface of hydrogel after the modification. This observation was emphasized through the EDX spectra of hydrogels. These results confirmed the successful modification of SH in the KOH solution. In addition, when observing the images of PF/SH and PF/SB, the images showed a significant change in surface morphology compared to SH and SB, respectively. This phenomenon is due to the existence of phosphate fertilizer adsorbed on the surface of the hydrogels, as demonstrated in the EDX mapping images of P element in Figure S1.

**Figure 4:**
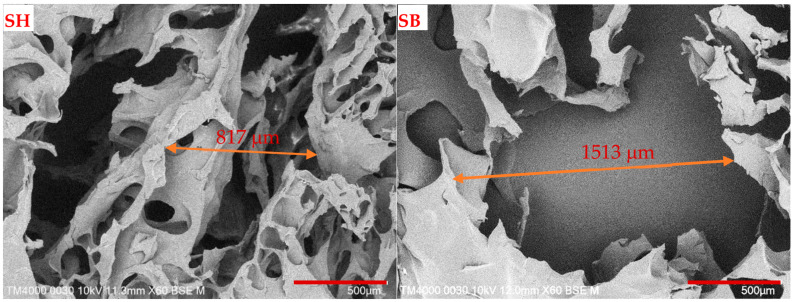
SEM images of SH and SB hydrogels.

SEM images of SH and SB in Figure 4 showed a uniform porous structure with the largest pore size and estimated porosity increased significantly after modification, with values of about 817 to 1513 µm and 49.95 to 61.09%, respectively. This result can be explained by the attack of hydroxyl ions on the longitudinal substituents on the polymer chains, cleaving the C–N bonds, forming –COO^–^ groups, and CH_3_–NH–CH_3_ molecules, as shown in Figure S2. Likely charged –COO^–^ groups could create repulsive forces and increase the distance between polymer chains. In addition, the appearance of hydrophilic groups –COO^–^ and the disappearance of hydrophobic methyl groups from the polymer increased the hydrophilicity of the hydrogel after modification, which led to the absorption of a greater amount of water and created larger pore sizes. The hydrolysis mechanism of amide groups in KOH can be described as follows. First, the nucleophile OH^−^ attacked the carbon atom of the C=O group and formed a tetrahedral intermediate. Then, the proton continued to attack the position of the –N(CH_3_)_2_ group and broke the C–N bond. Finally, the hydrolysis formed polycarboxylates and dimethylamine molecules [46].

**Figure 5:**
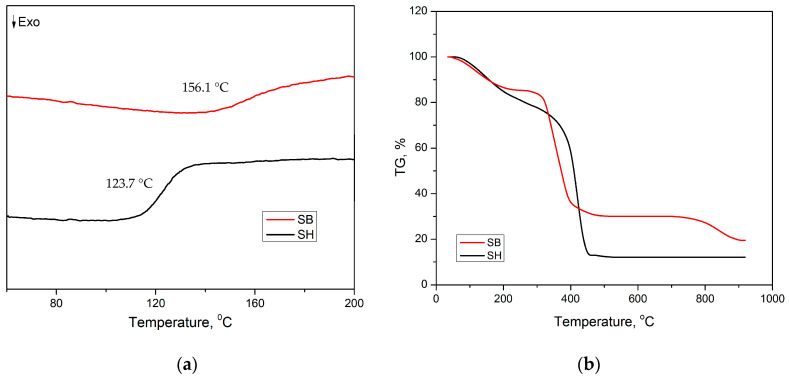
(**a**) DSC curves and (**b**) TGA curves of SH and SB hydrogels.

The glass transition temperature (T_g_) of the hydrogels was determined by DSC measurements. After the modification, Figure 5a shows the T_g_ increased significantly from 123.7 to 156.1 °C for SH and SB hydrogels, respectively. This could be explained by the formation of stable ionic bonds between the K^+^ and the carboxyl groups within the SB structure. These ionic bonds reduced the free movement of the polymer chains, so the SB hydrogel required higher energy to be able to perform the phase transition.

The impact of modification on the thermal stability of the hydrogel was evaluated by TGA measurement in the temperature range of 30–900 °C. Figure 5b shows that the weight loss of SH hydrogel started at a temperature of 56 °C, and then it continued to decline rapidly at 300 °C and stabilized at a remaining weight of 12.1% in the temperature range of 542–900 °C. These weight losses were attributed to the removal of water and the destruction of polymer chains [47]. For the SB hydrogel, there are three stages of thermal degradation, starting at temperatures at approximately 40, 270, and 700 °C. The first stage ended at 200 °C with a weight loss of about 14.7%, while the second stage ended at 400 °C with a remaining weight of about 30%, and in the final stage, the residual mass was 20% at 900 °C. The weight loss at the above stages also was attributed to the removal of water and the breaking of polymer chains [48]. The TGA results demonstrated the positive impact of the modification on the thermal stability of the hydrogel in this work.

**Figure 6:**
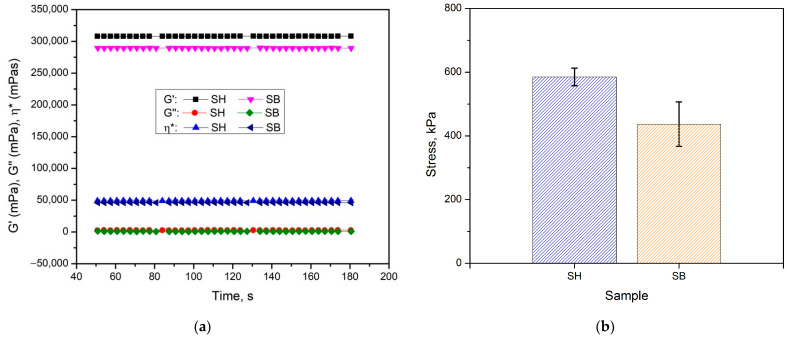
(**a**) The rheological and (**b**) compressive stress data of hydrogels.

The data of rheological and mechanical measurements are illustrated in Figure 6a,b, and summarized in Table 2. The viscoelastic behavior of the hydrogels was confirmed as the storage modulus (G′) values did not depend on time and were much higher than the loss modulus (G″) values [28]. In addition, the values of G′, G″, viscosity (η*), and compressive stress of SB were lower than those of SH, indicating a significant effect of the modification in the KOH solution. These results could be due to the hydrolysis of amide bonds, which created large pores and reduced the uniformity of hydrogel, leading to easier sliding of the polymer chains and reduced stress dissipation. This phenomenon was also completely consistent with SEM images, as discussed above.

### 3.2. Swelling Behavior

#### 3.2.1. The Impact of Salt Environments

**Figure 7:**
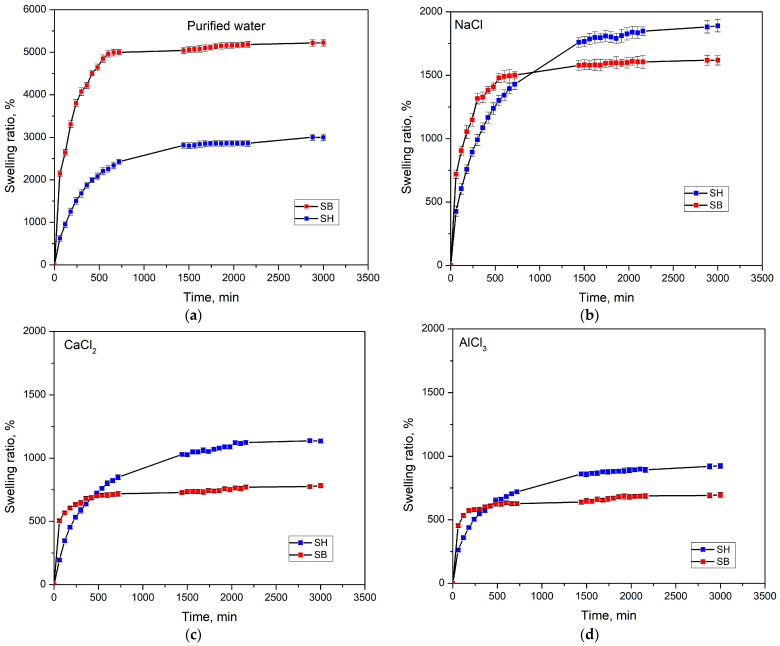
Swelling kinetic curves of hydrogels at 25 °C in (**a**) water and 50 mM solution of (**b**) NaCl, (**c**) CaCl_2_, (**d**) AlCl_3_.

The hydrogels synthesized in this work are aimed at applications in cultivation. The swelling of the hydrogels when present in the soil will determine its ability to be reused. Therefore, this behavior of the hydrogels in 50 mM salt solutions with metal ions of different valence was investigated. In addition, the swelling behavior in water was also performed for comparison. The results are presented in Figure 7, and the relevant swelling kinetic parameters are listed in Table 3. Figure 7a shows that the swelling rate of the hydrogels in water increased rapidly in the early stage and reached equilibrium after 600 min, in which the swelling rate and equilibrium swelling ratio (SR) of SB were superior to SH. This significant effect of modification on swelling behavior in water could be explained by the fact that during the modification, some links in the PAM were broken and formed shorter chains, reducing the folded structure, and the polymer chains tended to straighten more. This increased the surface area since the hydrophilic regions tend to be more outward, leading to increased swelling. In addition, the increase in pore size and porosity after modification with KOH (as discussed in the SEM images) could also have a significant impact on the swelling behavior of SB in water compared to SH hydrogels.

To evaluate the swelling properties of hydrogels in different salt environments, NaCl, CaCl_2_, and AlCl_3_ solutions at 25 °C were used. Figure 7b–d and Table 3 show that the SB hydrogel had a faster swelling rate and reached equilibrium in a shorter time. This is entirely because SB has a more favorable morphology than SH. In salt environments, SB has lower equilibrium SRs than SH, and these values have also gradually decreased with greater valence metal. Specifically, in NaCl, the SRs were 1889.43 ± 61.49, and 1617.93 ± 57.24%; in CaCl_2_, the SRs were 1136.74 ± 67.43, and 781.97 ± 43.24%; and in AlCl3, the SRs were 924.32 ± 48.77, and 695.79 ± 53.71% for SH and SB, respectively. This phenomenon could be explained by the presence of multivalent cations, such as Na^+^, Ca^2+^, and Al^3+^, which were able to exchange charges inside the gel by complexing with carboxamide or carboxylate groups. This leads to the formation of ionic bonds, resulting in increased crosslinking density and reduced space volume within the polymer structure, thereby significantly reducing the swelling capacity [49,50]. However, SB exhibited a lower SR than SH, possibly due to the existence of K^+^ ions after the modification, leading to increased osmotic pressure of the hydrogel. Thanks to the role of linear PAM, the hydrogel in this work has an equilibrium swelling time twice as fast as conventional hydrogels synthesized from the same materials, while SRs decreased insignificantly even though the amount of crosslinker increased up to sixteen times [26]. Compared to our previous reports, the hydrogels in this work had higher SR and a shorter time to reach equilibrium swelling [8,11].

The plots of t/S vs. t of SH and SB in water are shown in Figure S3, and the swelling kinetic parameters are presented in Table 3. The theoretical maximum swelling values (SR_max,the_) of SH, and SB were compatible with the obtained experimental data (SR_max,exp_). In addition, the diffusion exponent n, the specific rate constant k, and the diffusion coefficient D are listed in Table 3. The results showed that 0.07 ≤ n ≤ 0.39 (n ≤ 0.45), so it was possible to identify that the diffusion of water into the hydrogel obeyed the Fickian mechanism [51].

#### 3.2.2. Swelling Behavior in pH Solutions

**Figure 8:**
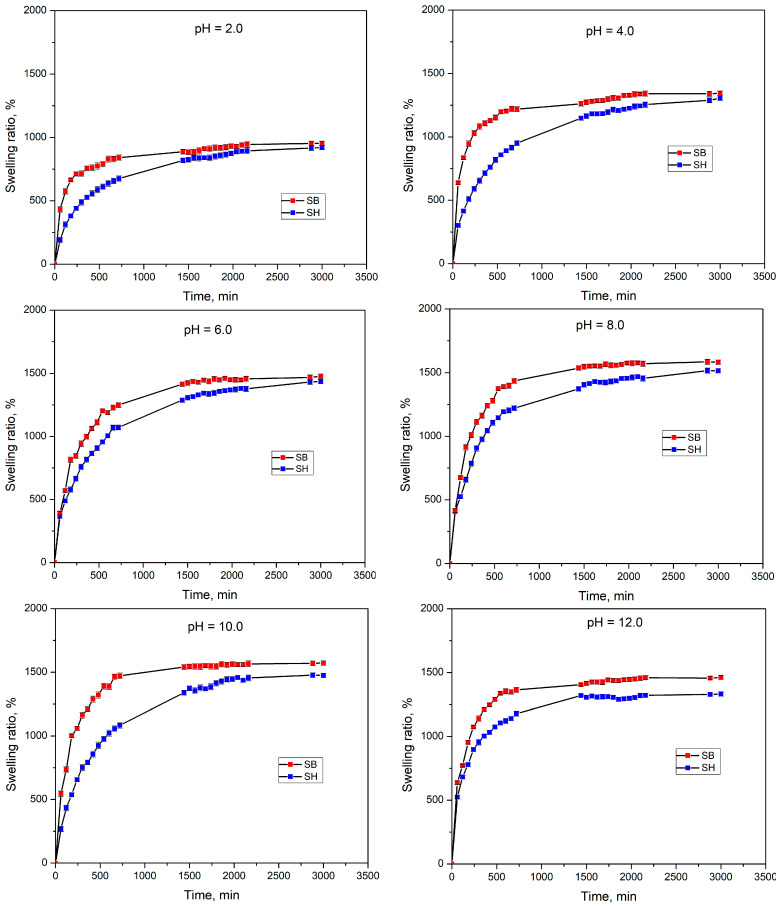
Swelling kinetic curves of hydrogels in pH solutions at 25 °C.

To evaluate the reusability of the hydrogels in acidified or alkaline soils, their swelling behavior in pH solutions was investigated. The swelling kinetic curves of hydrogels in pH2, pH4, pH6, pH8, pH10, and pH12 at 25 °C are shown in Figure 8, while the SR values are listed in Table 4. Under the same experimental conditions, SB had a higher swelling rate and a shorter time to reach equilibrium swelling than SH hydrogels. As is known, DMA-based hydrogels are not pH-sensitive due to the absence of ionizing groups [52]. However, the hydrogels in this work are pH sensitive after being copolymerized with MA [53]. At pH 2 (strong acid), the protonation of functional groups could be unfavorable leading to minimal water absorption. Then, SRs tended to increase as the pH values increased to pH 4, pH 6, and pH 8. This could be attributed to the ionization of functional groups, such as –N(CH_3_)_2_ to –NH(CH_3_)_2_]^+^ (for pH 4 and pH 6) causing cation-cation repulsion and –COOH to COO– (for pH 8) causing anion-anion repulsion. The repulsive forces between these ions expanded the structure of the hydrogels, enabling the absorption of larger amounts of water [54]. However, at pH > 8, the SRs reduced slightly due to the alkaline ions in the buffer solutions, reducing the electrostatic repulsion between the same charged –COO– groups. This phenomenon was also known as the “charge shielding effect” [50].

The plots of t/S vs. t of SH and SB in buffer solutions were also used to calculate swelling kinetic parameters, and the results are presented in Figure S4a–f and Table 4. The theoretical equilibrium swelling values (SR_max,the_) were consistent with the obtained experimental data. In addition, the plots of lnF vs. lnt of SH and SB in buffer pH solutions are shown in Figure S4g–l, while the diffusion exponent n, the specific rate constant k, and the diffusion coefficient D are listed in Table 4. The results showed that 0.14 ≤ n ≤ 0.36 (n ≤ 0.45), so it was possible to identify that the diffusion of water into the hydrogel also obeyed the Fickian mechanism [51].

### 3.3. Release of Phosphate Fertilizer

#### 3.3.1. Phosphate Fertilizer Sorption Behavior

**Figure 9:**
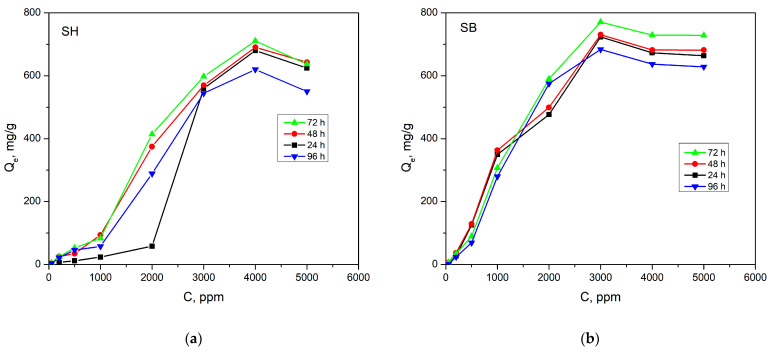
The amounts of loaded PF per mass of dried-hydrogel as a function of initial PF concentration of (**a**) SH, and (**b**) SB at 25 °C in 24, 48, 72, and 96 h.

The maximum wavelength absorption of phosphate fertilizer (PF) is 880 nm [36]. A series of PF solutions with concentrations ranging from 2 to 20 ppm were prepared to develop the calibration curve with R^2^ = 0.9992. The amounts of loaded PF per mass of dried-hydrogel (Q_e_) of SH and SB at initial concentrations of 50–5000 ppm in 24, 48, 72, and 96 h at 25 °C are shown in Figure 9. When the initial PF concentration and contact time increased to a certain critical value, the Q_e_ increased correspondingly. For SH, Q_e_ reached a maximum of 710.8 mg/g at 4000 ppm after 72 h, while it was 770.9 mg/g at 3000 ppm after also 72 h for SB. This demonstrated a significant influence of hydrogel morphology on PF absorption. Due to the larger pore size and porosity, PF adsorption onto SB may have been more favorable, requiring a lower concentration gradient compared to SH. However, when the initial PF concentration exceeded the critical values, reverse osmosis caused Q_e_ to decrease.

#### 3.3.2. Phosphate Fertilizer Release Behavior

**Figure 10:**
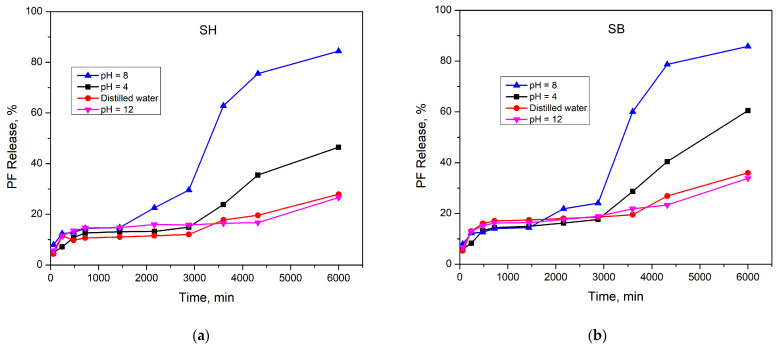
Release behavior of PF at 25 °C in pH 4, pH 8, pH 12, and distilled water of (**a**) PF/SH and (**b**) PF/SB.

To evaluate how alkaline or acidified environments affect PF release behavior, the PF release from PF-loaded hydrogels was investigated at three different pHs, pH 4, pH 8, and pH 12, at a temperature of 25 °C. Figure 10a,b shows the cumulative PF release amount gradually increased with time. For SH, the % release (RP) within 100 h was 46.47%, 84.42%, and 26.62% at pH 4, pH 8, and pH 12, respectively, while these values for SB were 60.50%, 85.80% and 33.85%, respectively. This result confirmed that the modification changed the morphology, leading to changes in the release properties of the hydrogels.

**Figure 11:**
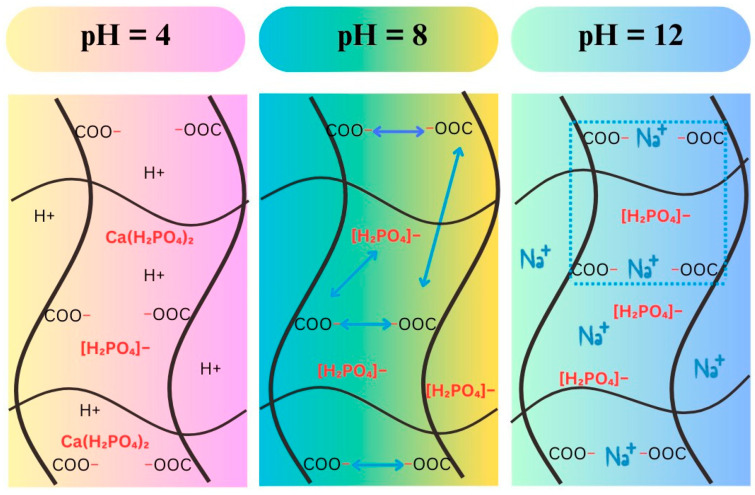
The structures of PF/hydrogels at pH 4, pH 8 and pH 12.

On the other side, the release speed was slow and did not change significantly in all three pH environments within the first 3000 min. And then, this speed increased significantly at pH 4 and pH 8, while this phenomenon was not observed at pH 12. This result may be due to at the initial stage, the dried hydrogels need time to become the swollen state. When the appropriate swelling state has not been reached, most of the PF will be retained inside the hydrogel. The PF release at pH 12 was the lowest due to the charge shielding effect, as discussed above. The existence of a high concentration of Na^+^ ions prevented the repulsion between –COO– ions, which could have significantly changed the morphology of the hydrogel. Additionally, these Na^+^ ions may have interacted with the H_2_PO_4_^–^ molecules, leading to the PF being entrapped inside the material. At pH 8, the PF release was the highest. This result is because in this environment the charge shielding effect did not occur; the –COO– groups created a strong repulsive force for each other, causing structural expansion that facilitated the release of PF. Finally, in the acidic environment of pH 4, the dissociation of Ca(H_2_PO_4_)_2_ ⇌ Ca^2+^ + 2H_2_PO_4_^–^ may have been limited, so the pF release speed and efficiency were lower than that at pH 8. The structures of PF/hydrogels at pH 4, pH 8, and pH 12 are illustrated in Figure 11.

To evaluate the PF release mechanism and the factors that influence this process, four kinetic models were applied to confirm the best-fit model, which includes the zero-order kinetic model (Z–O), first-order kinetic model (F–O), Higuchi (H), and Korsmeyer–Peppas model (K–P). The release kinetics of SH and SB at pH 4, pH 8, and pH 12 are listed in Table 5. As usual, the correlation coefficient R^2^ is the most important factor in determining the best-fit model. At pH 4 and pH 8, the PF release of the hydrogels followed the F–O model, meaning that the main factors driving this process were diffusion, surface area, and concentration gradients [55], and at pH 12, the PF release behavior followed the K–P model. In addition, the two n values in the K–P model were both less than 0.45, showing that the PF release at pH 12 was consistent with the Fickian diffusion mechanism [55].

### 3.4. Impact of Hydrogels on Soil Properties

**Figure 12:**
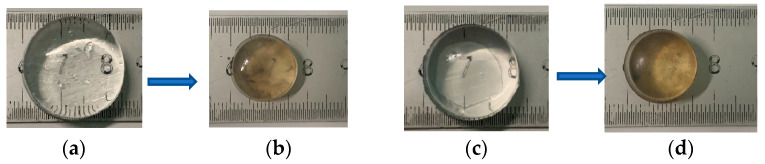
Photograph images of the equilibrium swelling hydrogels: (**a**) SB before the first cycle; (**b**) SB after the sixth cycle; (**c**) SH before the first cycle; and (**d**) SH after the sixth cycle.

The ability of the hydrogel to provide moisture was evaluated by measuring soil moisture as a function of time. The results of the six experimental cycles are depicted in Figure S5. In all six cycles, the moisture content of SB/soil was always higher than the SH/soil, and these values tended to decrease from about 45% to about 20% on day 0 and day 12, respectively. After six cycles of water absorption and release, the soil moisture only decreased slightly from 45.92% (day 0, cycle 1) to 44.28% (day 0, cycle 6) and from about 20% (day 12, cycle 1) down to 19.07% (day 12, cycle 6). The reusability of hydrogels was also determined by their appearance. Photograph images of the equilibrium swelling hydrogels before and after the experiments are presented in Figure 12. The results showed that, after six experimental cycles, both SH and SB hydrogels remained intact, with no cracking phenomenon, and the equilibrium swelling diameter of both hydrogels decreased from 26.1 to 19.7 mm, and from 21.9 to 16.3 mm for SB and SH, respectively. This demonstrated that the mechanical properties of the hydrogel in this work met the requirements and ensured feasibility for further applications. The decrease in size may be due to the absorption of ions by the hydrogels that were present in the soil. The results of reuse experiments also showed the economic viability of obtained hydrogels. Finally, in all cases, the swelling equilibrium size of SB was always higher than that of SH. This is completely consistent with the conclusion that SB has superior swelling ability than SH as discussed above.

As is known, soil acidification or alkalinization both have negative effects on plant growth. To evaluate the impact of hydrogels on soil pH value, the pH of SH/soil and SB/soil was continuously measured during six experimental cycles. Figure S6 shows that the pH values only changed within a very small range of 7.498–7.698. This marginal difference indicated a negligible effect of the hydrogels on soil quality.

From the above results, the high reusability of hydrogels, which was attributed to their stable three-dimensional structure, was demonstrated. It could be concluded that the hydrogels in this work were suitable choices for the release of moisture and PF applications in soil.

### 3.5. Plant Growth Performance

**Figure 13:**
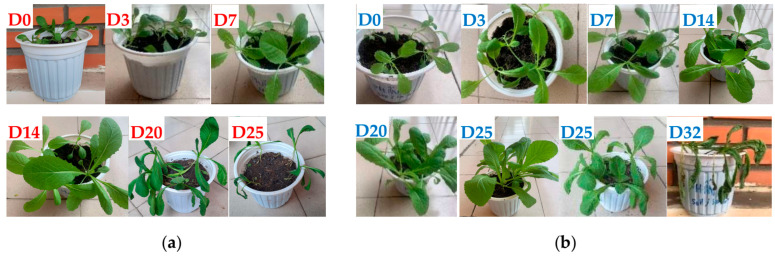
Growth of mustard greens in: (**a**) PF/SH/Soil after 25 days, and (**b**) PF/SB/Soil after 32 days.

The applicability of hydrogels in agricultural technology was evaluated by observing the growth of mustard greens grown in soil containing PF/SH and PF/SB hydrogels. Figure 13 shows the growth of mustard greens in soil containing PF/SH observed for 25 days, and containing PF/SB observed for 32 days. The results showed that the leaves were wilted after 20 days when placed in soil containing PF/SH, while this phenomenon could be only observed after 32 days for soil containing PF/SB. To evaluate the impact of hydrogels on the growth process of mustard greens, the leaf length as a function of time was measured. Table 6 shows that the leaf length of mustard greens in PF/SB/Soil was a little longer than that in PF/SH/Soil. This difference was more obvious from day 7 to day 20. Finally, despite not adding water, the plants only stopped growing on day 20 for SH, and day 32 for SB hydrogel. These results may be because mustard greens are short-day plants with large foliage, so they need a lot of water and nutrients in a short time. SB hydrogel met these requirements as shown by the results discussed above regarding absorption and release of moisture and PF.

## 4. Conclusions

This study reported on semi-IPN hydrogels using PAM, DMA, and MA as raw materials. The obtained semi-IPN hydrogel was modified in a KOH solution. FTIR spectra confirmed the successful polymerization, modification, and loading of PF into the hydrogels. The uniform porous structure and the increase in the largest pore size of hydrogels from 817 to 1513 µm after modification were demonstrated by SEM images. In general, the modification of semi-IPN hydrogel with KOH solution had positive effects on the thermal properties, mechanical properties, and especially the swelling behavior in different environments, such as water or pH buffer solutions, when SR was increased up to 151% in water. After modification, the loaded PF per mass of dried hydrogels increased from 710.8 mg/g to 770.9 mg/g. The pH environment had a significant influence on the PF release behavior of the hydrogel with the % release reaching a maximum at pH 8 of 85.8%. On the other side, at pH 4 and pH 8, the PF release behavior of hydrogels followed the F–O model, while at pH 12 this behavior followed the K–P model and was consistent with the Fickian diffusion mechanism. The economic viability of the obtained hydrogels in this work was also demonstrated as the hydrogels were stable in the soil after six cycles of water absorption and release experiments, and their use did not cause acidification or alkalinization. Finally, the modification of the hydrogel increased the lifespan of mustard greens from 20 to 32 days. These results showed the potential applications of modified semi-IPN hydrogel materials in cultivation shortly.

## Figures and Tables

**Table 1:** The mathematical equation of PF release models.

Model	Mathematical Equation	
Zero-order (Z–O)	$C_{r}=C_{0}-k_{o}t$	(8)
First-order (F–O)	$\ln C_{r}=\ln C_{o}-k_{1}t$	(9)
Higuchi (H)	$\frac{C_{r}}{C_{\infty }}=k_{H}\sqrt{t}$	(10)
Korsmeyer–Peppas (K–P)	$\ln\frac{C_{r}}{C_{\infty }}=\ln k_{KP}-n\ln t$	(11)

where C_r_ (mg/L) is a concentration of PF release in time t; C_o_ (mg/L)is the initial concentration of PF in the solution; k_o_ (mg/(L.h)) is the zero-order release constant; t (h) is time; k_1_ (1/h) is the first-order release constant; C_∞_ (mg/L) is a concentration of PF release in equilibrium; k_H_ (1/h^1/2^) is the Higuchi release rate constant; k_KP_ is Korsmeyer–Peppas release rate constant; n is release exponent.

**Table 2:** Data on rheological and mechanical measurements of hydrogels.

Hydrogel	Storage Modulus, G′ (Pa)	Loss Modulus, G″ (Pa)	Viscosity, η* (Pa·s)	Compressive Stress (kPa)
SH	410.6 ± 10.9	6.2 ± 0.1	66.4 ± 1.7	585.8 ± 52.8
SB	273.6 ± 9.8	0.8 ± 0.1	43.5 ± 1.6	436.7 ± 69.9

**Table 3:** Swelling kinetic parameter of hydrogel in salt solution at 25 °C.

		SR_max,exp_, %	SR_max,the_, %	k_s_	n	k	D	R^2^
H_2_O	SH	3450.22 ± 43.56	3984.06	7.11 × 10^−5^	0.39	0.054	1.68 × 10^−5^	0.9478
	SB	5222.11 ± 68.78	5276.34	2.15 × 10^−4^	0.18	0.245	5.96 × 10^−5^	0.8018
NaCl	SH	1889.43 ± 61.49	2109.70	6.86 × 10^−4^	0.33	0.080	2.86 × 10^−5^	0.9618
	SB	1617.93 ± 57.24	1669.45	1.50 × 10^−4^	0.16	0.300	6.97 × 10^−7^	0.8500
CaCl_2_	SH	1136.74 ± 67.43	1282.05	5.22 × 10^−4^	0.34	0.078	3.00 × 10^−5^	0.9536
	SB	781.97 ± 43.24	781.25	1.27 × 10^−4^	0.08	0.531	8.38 × 10^−11^	0.9125
AlCl_3_	SH	924.32 ± 48.77	998.00	4.08 × 10^−4^	0.26	0.142	7.88 × 10^−6^	0.9671
	SB	695.79 ± 53.71	700.28	1.14 × 10^−4^	0.07	0.560	1.71 × 10^−11^	0.9139

**Table 4:** Swelling kinetic parameter of hydrogels in pH solutions at 25 °C.

		SR_max,exp_	SR_max,the_	k_s_	n	k	D	R^2^
pH = 2	SH	920.51 ± 14.69	1008.06	3.05 × 10^−4^	0.30	0.101	1.69 × 10^−5^	0.9695
	SB	953.70 ± 13.30	978.47	9.08 × 10^−4^	0.14	0.333	1.95 × 10^−5^	0.9171
pH = 4	SH	1331.35 ± 22.03	1396.65	5.47 × 10^−4^	0.19	0.233	1.54 × 10^−6^	0.9216
	SB	1461.85 ± 20.19	1508.30	7.50 × 10^−4^	0.16	0.295	8.19 × 10^−5^	0.8378
pH = 6	SH	1435.56 ± 22.36	1584.79	1.98 × 10^−4^	0.31	0.094	1.90 × 10^−5^	0.9763
	SB	1477.69 ± 20.32	1715.27	2.82 × 10^−4^	0.26	0.134	1.91 × 10^−5^	0.9051
pH = 8	SH	1516.46 ± 17.89	1628.66	2.58 × 10^−4^	0.28	0.117	1.33 × 10^−5^	0.9316
	SB	1585.08 ± 20.02	1680.67	4.07 × 10^−4^	0.24	0.163	1.34 × 10^−5^	0.8419
pH = 10	SH	1477.08 ± 15.79	1692.05	1.57 × 10^−4^	0.36	0.066	3.89 × 10^−5^	0.9633
	SB	1572.22 ± 15.36	1647.45	5.35 × 10^−4^	0.20	0.222	4.34 × 10^−5^	0.8445
pH = 12	SH	1304.72 ± 20.39	1440.92	2.01 × 10^−4^	0.32	0.087	2.16 × 10^−5^	0.9782
	SB	1344.59 ± 19.19	1377.41	8.11 × 10^−4^	0.14	0.351	2.46 × 10^−5^	0.8765

**Table 5:** Kinetic parameters of PF release.

Model		SH			SB		
		pH = 4	pH = 8	pH = 12	pH = 4	pH = 8	pH = 12
Z–O	C_o_	9.9259	6.3337	20.8530	9.1705	4.7774	20.9525
	k_o_	−0.0144	−0.0282	−0.0046	−0.0164	−0.0301	−0.0065
	R^2^	0.8939	0.9104	0.7466	0.8835	0.8716	0.8692
F–O	C_o_	16.4233	19.9355	20.8927	17.3744	20.3443	21.8828
	k_1_	−3 × 10^−4^	−4 × 10^−4^	−2 × 10^−4^	−3 × 10^−4^	−4 × 10^−4^	−2 × 10^−4^
	R^2^	0.9041	0.9307	0.5969	0.8964	0.9140	0.7128
H	k_H_	0.0110	0.0134	0.0073	0.0106	0.0132	0.0085
	R^2^	0.7938	0.8117	0.7543	0.7424	0.7492	0.8374
K–P	n	0.4280	0.5116	0.2443	0.4332	0.5040	0.2736
	K_KP_	0.0155	0.0078	0.0956	0.0137	0.0079	0.0729
	R^2^	0.8407	0.7824	0.8308	0.8087	0.7177	0.8786

**Table 6:** Leaf length (cm) of mustard greens as a function of time.

Days	0	3	7	14	20	25	30
PF/SH/Soil	3.5 ± 0.11	5.3 ± 0.22	6.2 ± 0.19	12.8 ± 0.42	14.2 ± 0.46	-	-
PF/SB/Soil	3.5 ± 0.14	5.6 ± 0.17	6.9 ± 0.21	13.6 ± 0.43	15.3 ± 0.49	17.2 ± 0.54	17.6 ± 0.55

## Data Availability

All data and materials are available on request from the corresponding author. The data are not publicly available due to ongoing research using a part of the data.

## Supplementary Materials

The following supporting information can be downloaded at: https://www.mdpi.com/article/10.3390/polym16091195/s1, Figure S1: EDX mapping image of P element in (a) PF/SH, and (b) PF/SB hydrogel; Figure S2: Mechanism of hydrolysis of the amide group in KOH; Figure S3: (a–d) the plot of t/S vs. t, and (e–h) the plot of lnF vs. lnt of hydrogels in water and salt environments; Figure S4: (a–f) the plot of t/S vs. t, and (g–l) the plot of lnF vs. lnt of hydrogels in pH buffer solutions. Figure S5: The moisture content of soil/SH and soil/SB as a function of time over six experimental cycles. Figure S6: pH values of soil/SH and soil/SB as a function of time over six experimental cycles.
