# Supplementary material for: Impact of Modifications from Potassium Hydroxide on Porous Semi-IPN Hydrogel Properties and Its Application in Cultivation
Source: Polymers (Basel). 2024 Apr 25;16(9):1195. doi: 10.3390/polym16091195 (PMC11085908; doi:10.3390/polym16091195)
Supplement: Supplementary file 1 [file polymers-16-01195-s001.zip › polymers-2943088-supplementary.pdf]

Supplementary Materials: Impact of modification in potassium hydroxide on porous semi-IPN hydrogel properties and its application in cultivation

Huynh Nguyen Anh Tuan[1*], Bui Thi Cam Phan[1], Ha Ngoc Giang[2], Giang Tien Nguyen[1], Thi Duy Hanh Le[1], Ho Phuong[1]

1 Faculty of Chemical and Food Technology, Ho Chi Minh City University of Technology and Education, No. 1, Vo Van Ngan Street, Linh Chieu Ward, Thu Duc, Ho Chi Minh City 71307, Vietnam
2 Faculty of Chemical Technology, Ho Chi Minh City University of Industry and Trade, No. 140, Le Trong Tan Street, Tay Thanh Ward, Tan Phu District, Ho Chi Minh City 72009, Vietnam
* Correspondence: tuanhna@hcmute.edu.vn

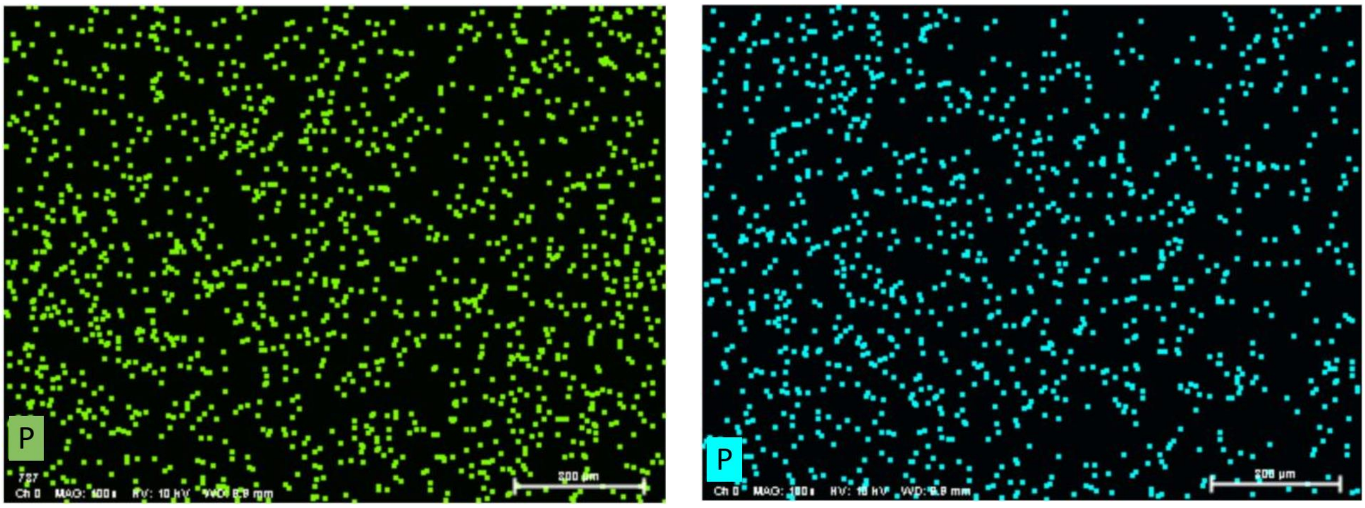

(a) **(b)**

Figure S1. EDX mapping image of P element in (a) PF/SH, and (b) PF/SB hydrogel

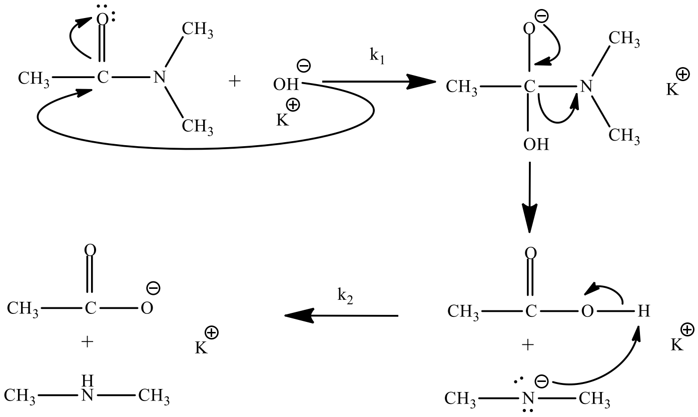

Figure S2. Mechanism of hydrolysis of the amide group in KOH.

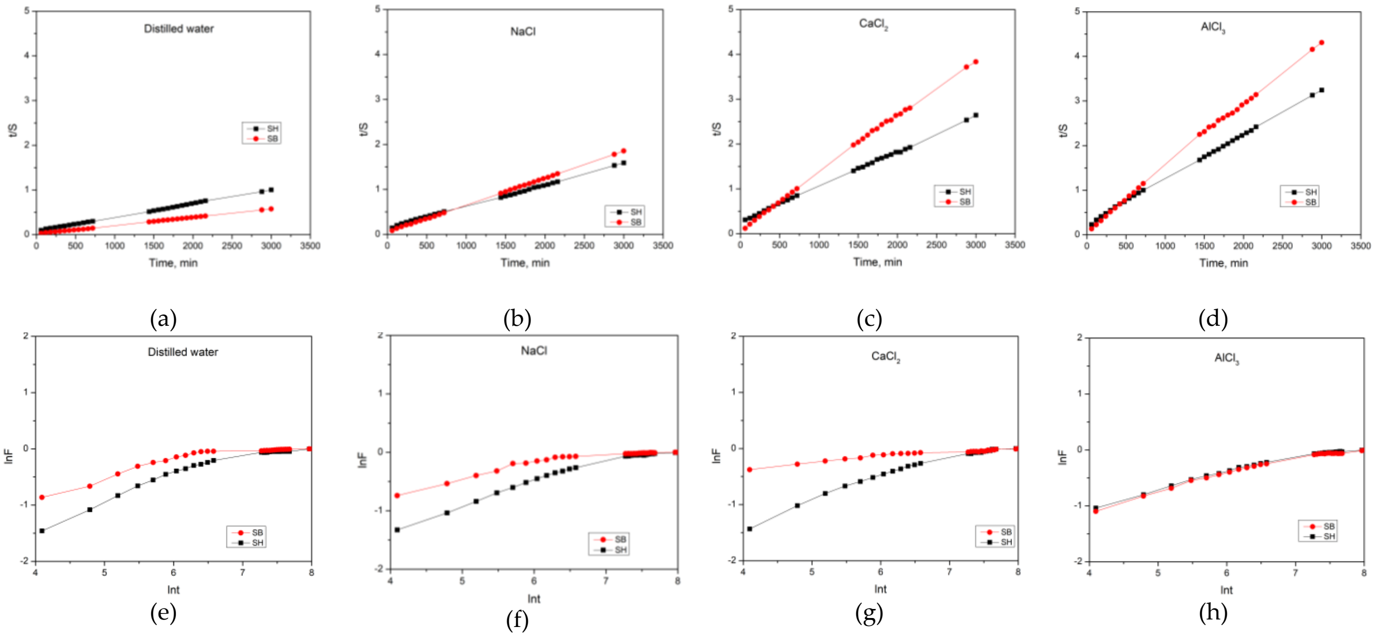

Figure S3. (a-d) the **p**lot of t/S vs. t, and (e-h) the plot of lnF vs. lnt of hydrogels in water and salt environments.

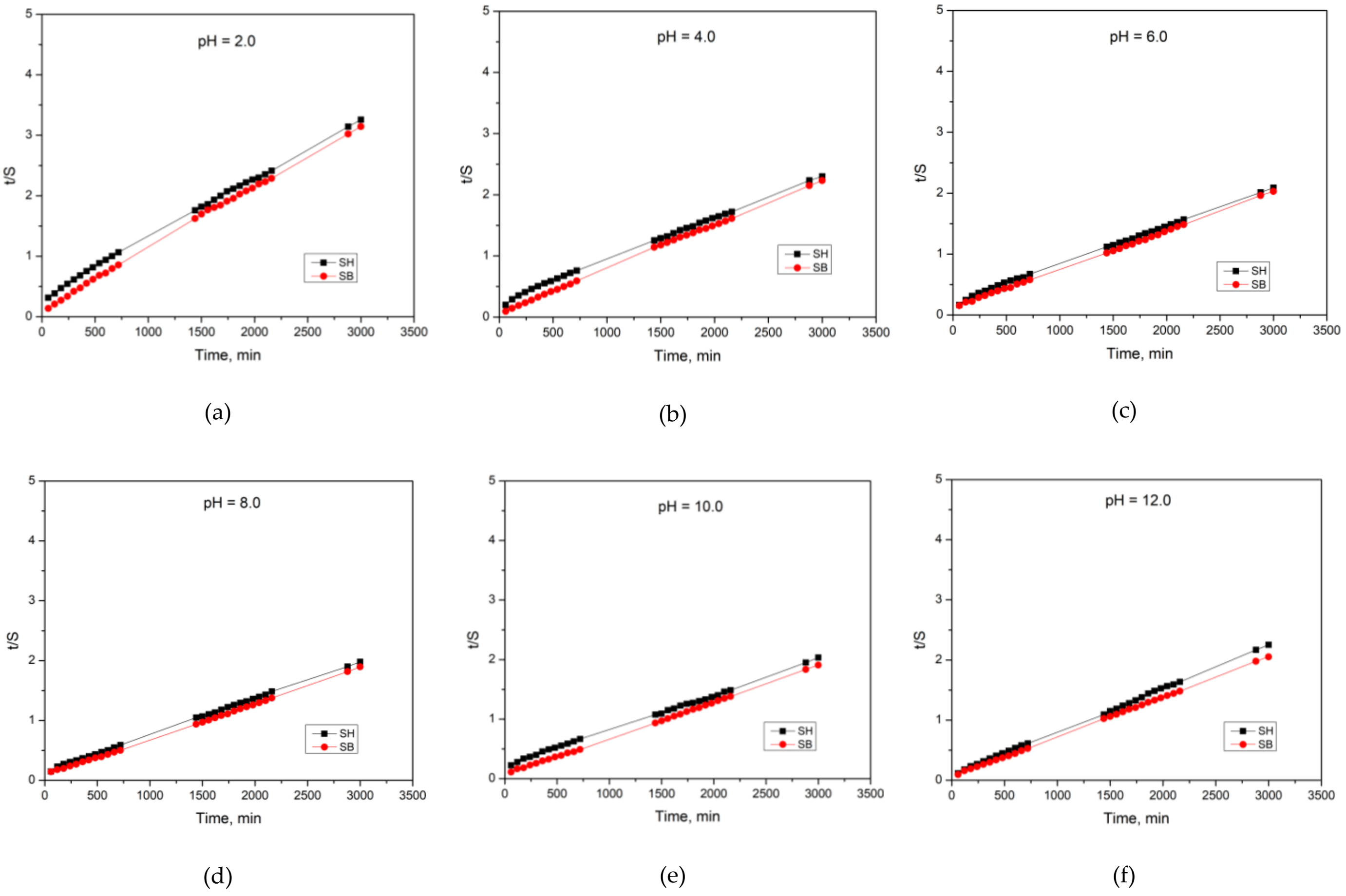

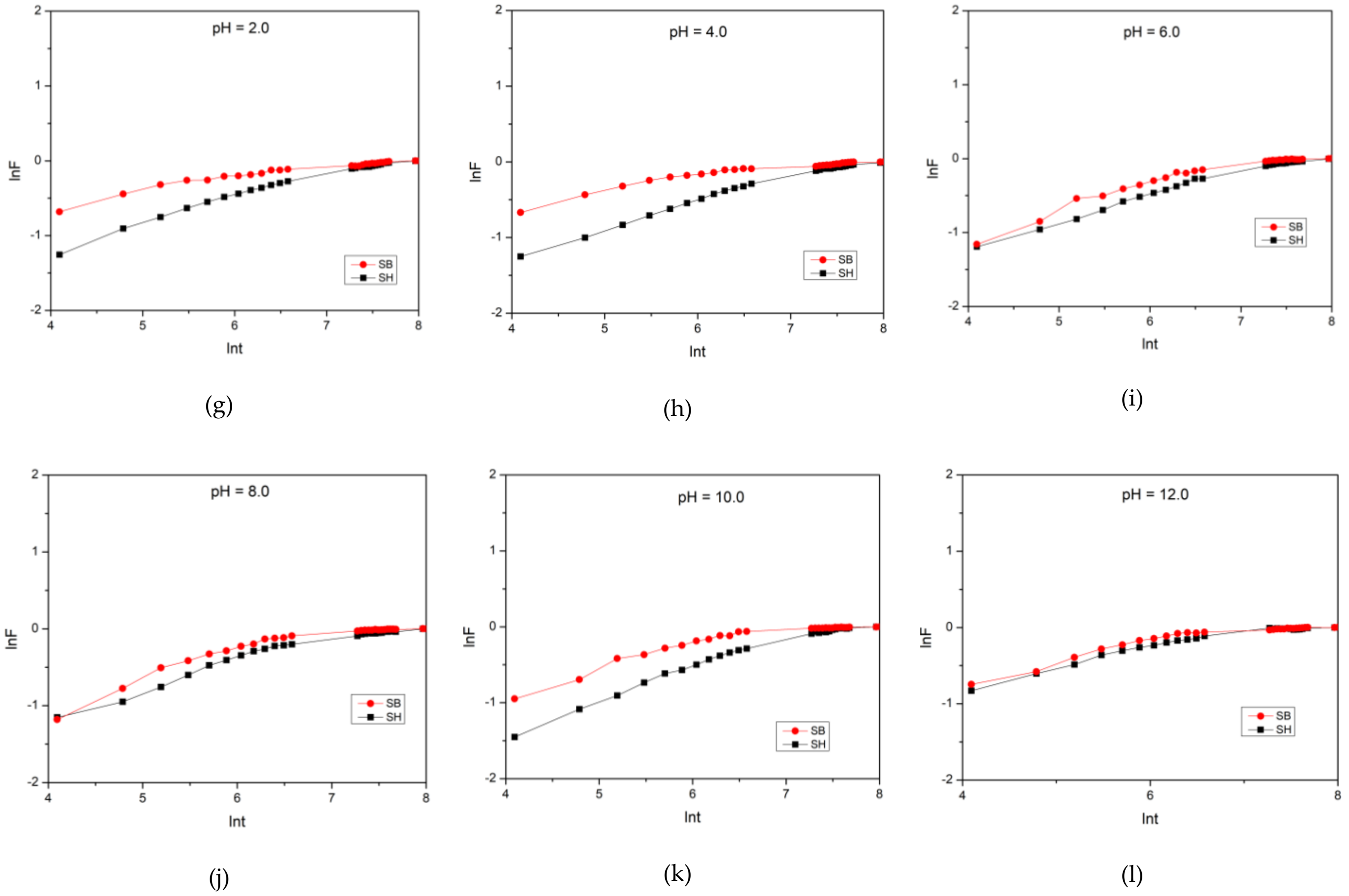

Figure S4. (a-f) the plot of t/S vs. t, and (g-l) the plot of lnF vs. lnt of hydrogels in pH buffer solutions.

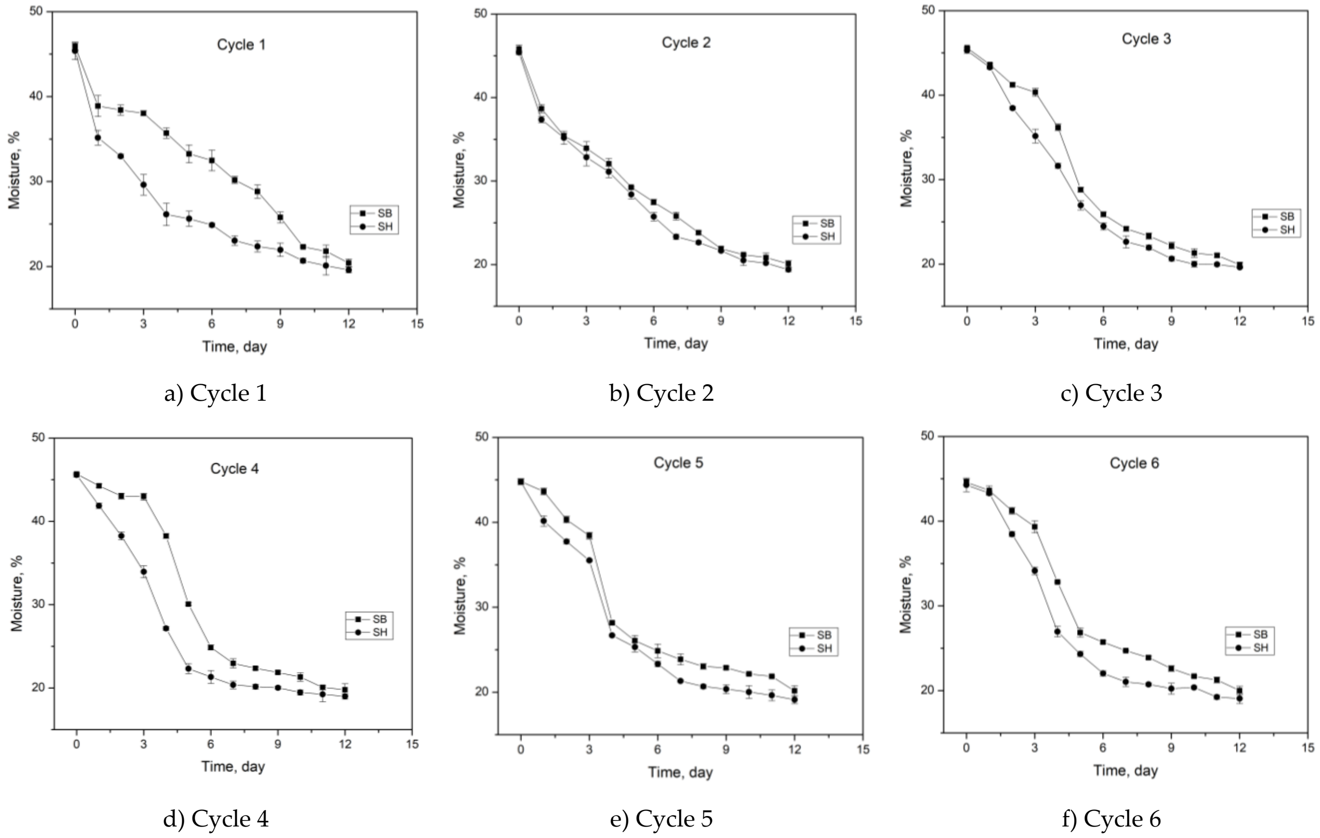

Figure S5. The moisture content of soil/SH and soil/SB as a function of time over six experimental cycles.

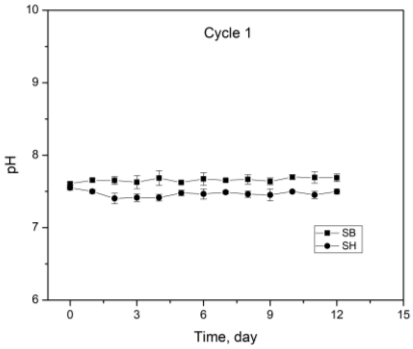

a) Cycle 1

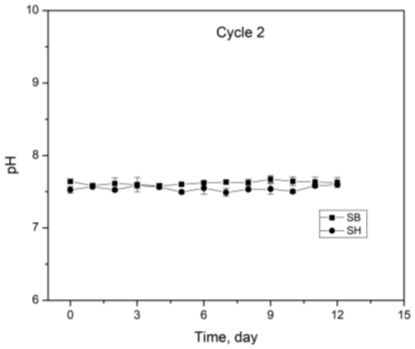

b) Cycle 2

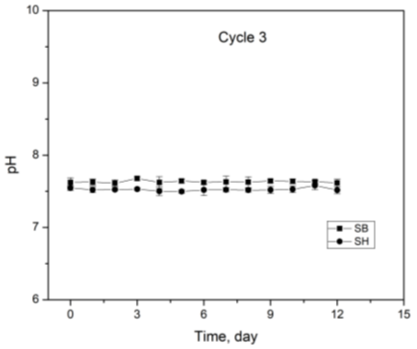

c) Cycle 3

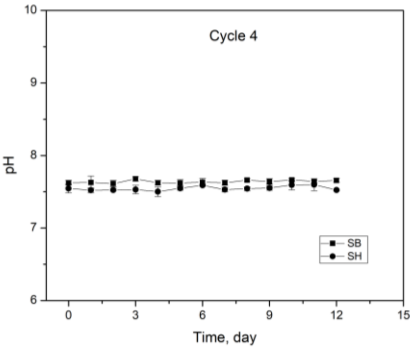

d) Cycle 4

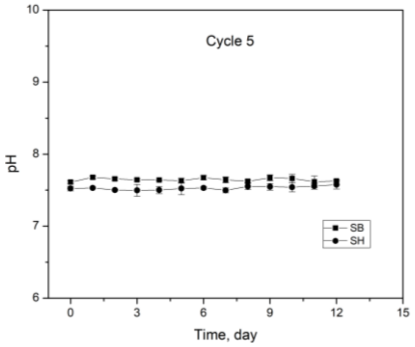

e) Cycle 5

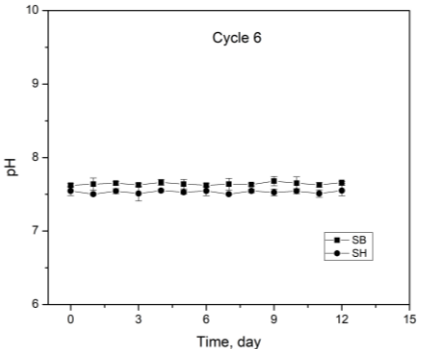

f) Cycle 6

Figure S6. pH values of soil/SH and soil/SB as function of time over six experimental cycles.
